# Is rotavirus aetiology in young children with acute diarrhoea associated with sociodemographic and clinical factors, including rotavirus vaccination status? A secondary cross-sectional analysis of the ABCD trial

**DOI:** 10.1136/bmjgh-2024-018337

**Published:** 2025-07-27

**Authors:** Sarah Somji, Christopher R Sudfeld, Christopher Duggan, Karim Manji, Tahmeed Ahmed, Mohammod Jobayer Chisti, Usha Dhingra, Sunil Sazawal, Benson Singa, Judd L Walson, Patricia B Pavlinac, Naor Bar-Zeev, Eric Houpt, Queen Dube, Karen L Kotloff, Samba O Sow, Mohammad Tahir Yousafzai, Farah Naz Qamar, Rajiv Bahl, Ayesha De Costa, Jonathon L Simon, Per Ashorn, ABCD Study Group, Muhammad Waliur Rahman

**Affiliations:** 1 Department of Epidemiology and Biostatistics, Muhimbili University of Health and Allied Sciences, Dar es Salaam, Tanzania, United Republic of; 2 Center for Child, Adolescent, Maternal Health Research, Faculty of Medicine and Health Technology, Tampere University and Tampere University Hospital, Tampere University and Tampere University Hospital, Tampere, Finland; 3 Departments of Global Health and Population and Nutrition, Harvard School of Public Health, Boston, Massachusetts, USA; 4 Division of Gastroenterology, Hepatology and Nutrition, Boston Children’s Hospital, Boston, Massachusetts, USA; 5 Departments of Global Health and Population and Nutrition, Harvard T H Chan School of Public Health, Boston, Massachusetts, USA; 6 Department of Paediatrics and Child Health, Muhimbili University of Health and Allied Sciences, Dar es Salaam, Tanzania, United Republic of; 7 Nutrition and Clinical Services Division, International Centre for Diarrhoeal Disease Research Bangladesh, Dhaka, Bangladesh; 8 Nutrition and Clinical Services Division, International Centre for Diarrhoeal Disease Research, Dhaka, Bangladesh; 9 Center for Public Health Kinetics, New Delhi, New Delhi, India; 10 Center for Clinical Research, Kenya Medical Research Institute, Nairobi, Kenya; 11 Department of Global Health and Epidemiology, University of Washington, Seattle, Washington, USA; 12 Department of Pediatrics and Medicine (Infectious Diseases), University of Washington, Seattle, Washington, USA; 13 Departments of Global Health, University of Washington, Seattle, Washington, USA; 14 Department of Epidemiology, University of Washington, Seattle, Washington, USA; 15 International Vaccine Access Center, Department of International Health, Johns Hopkins University Bloomberg School of Public Health, Baltimore, Maryland, USA; 16 Department of Medicine, Infectious Diseases, International Health, University of Virginia, Charlottesville, Virginia, USA; 17 Department of Pediatrics, Queen Elizabeth Central Hospital, Blantyre, Southern Region, Malawi; 18 Center for Vaccine Development and Global Health, University of Maryland Baltimore School of Medicine, Baltimore, Maryland, USA; 19 Department of Pediatrics, University of Maryland Baltimore School of Medicine, Baltimore, Maryland, USA; 20 Centre pour le Développement des Vaccines, Bamako, Mali; 21 Department of Pediatrics and Child Heath, The Aga Khan University, Karachi, Pakistan; 22 Department of Maternal, Child, and Adolescent Health and Aging, World Health Organization, Geneva, Switzerland; 23 Department of Maternal, Newborn, Child and Adolescent Health, and Ageing, World Health Organization, Geneve, Switzerland; 24 Center for Child, Adolescent, Maternal Health Research, Faculty of Medicine and Health Technology, Tampere University, Tampere, Finland

**Keywords:** Global Health, Child health, Vaccines, Cross-sectional survey

## Abstract

**Introduction:**

One of the leading causes of global child mortality continues to be diarrhoea where rotavirus contributed to about 24% of deaths among all diarrhoeal deaths, mostly in low-income and middle-income countries. Rotavirus vaccination programmes have contributed to the reduction of mortality from 24% to 19% in rotavirus infections among hospitalised children, but the burden of rotaviral diarrhoea remains high, especially in settings with undernutrition. We aimed to determine the association of rotaviral diarrhoea aetiology with prior vaccination, socioeconomic status and clinical factors in children to see their utility in clinical settings.

**Methods:**

We analysed secondary data from a multicentre clinical trial on antibiotic impact in children with diarrhoea and increased risk of mortality. We used stored stool samples of 6697 children aged 2–23 months old, presenting to a health facility with diarrhoea and increased risk of mortality. We determined rotavirus aetiology prevalence using quantitative PCR (qPCR) and looked at its association with the patient’s rotaviral vaccination status, clinical symptoms and sociodemographic characteristics. Prevalence ratios (PR) were calculated with log-binomial regression models; if they did not converge, log-Poisson models were used.

**Results:**

Rotavirus prevalence of 21.1% was observed. There was a weak and statistically non-significant inverse association between rotavirus vaccination and rotaviral diarrhoea aetiology (adjusted PR: 0.71, 95% CI 0.49 to 1.03). Of the five tested clinical symptoms, shorter diarrhoea duration was associated with rotaviral aetiology (PR: 2.65; 95% CI: 1.29 to 5.45). Of the seven tested socioeconomic characteristics, only maternal and paternal secondary education compared with no formal education were associated with rotaviral aetiology (PR: 0.86; 95% CI: 0.74 to 1.00, PR: 0.87, 95% CI: 0.75 to 1.00 respectively).

**Conclusion:**

Rotaviral diarrhoea aetiology cannot accurately be determined with prior receipt of rotavirus vaccination among children presenting to facilities with diarrhoea and increased risk of mortality. Short diarrhoea duration and parental secondary education were associated with increased prevalence of rotaviral aetiology; however, their utility in clinical care remains unclear.

**WHAT IS ALREADY KNOWN ON THIS TOPIC**

- Rotavirus vaccination has reduced rotavirus-associated diarrhoea mortality from 24% to 19% in hospitalised children with rotavirus infections.
- Despite vaccination, rotavirus diarrhoea continues to remain high, about 24% of deaths among all diarrhoeal deaths specifically in settings with undernutrition.

**WHAT THIS STUDY ADDS**

- Rotavirus vaccination had a statistically non-significant inverse association with rotavirus diarrhoea aetiology.
- Shorter diarrhoea duration compared with longer diarrhoea duration was associated with higher prevalence of rotaviral aetiology.
- Maternal and paternal secondary education compared with no formal education was associated with lower prevalence of rotaviral aetiology.

**HOW THIS STUDY MIGHT AFFECT RESEARCH, PRACTICE OR POLICY**

- Using information like the child’s earlier vaccination or other risk factors of rotaviral diarrhoea aetiology like shorter diarrhoea duration and maternal and paternal education is not very useful in assisting clinical staff in identifying children with rotaviral diarrhoea among all those presenting with diarrhoea at a health facility.

## Introduction

 Diarrhoea remains one of the leading causes of global child mortality and was estimated to cause about 444 000 child deaths in 2021.^1^ In 2013, prior to the roll-out of vaccination programmes, rotavirus infection was responsible for about 24% of diarrhoeal deaths in childhood, most of which occurred in low and middle-income countries (LMICs).^2 3^ Several countries in East and Southern Africa have implemented rotavirus vaccine roll-outs, with coverage increasing from 0% in 2010 to 90% in 2015. Concomitantly, there is evidence that vaccination has contributed to a reduction in rotavirus infections among children hospitalised with diarrhoea.^4^ Rotavirus vaccination is also associated with a reduced rate of rotavirus infection, especially in subpopulations of children such as those admitted with undernutrition.^5^ However, despite rotavirus vaccination programmes, the burden of rotaviral infection remains high, with rotaviral diarrhoea estimated to account for 19% of diarrhoeal deaths globally in 2019.^6^

Even though rotavirus vaccination reduces the incidence of rotavirus-associated diarrhoea, the numbers of cases remain high in LMICs.^3^ Several studies indicate that rotavirus vaccine is effective for severe rotavirus gastroenteritis.^4 7^ In a randomised controlled trial, 1.9% of infants in the rotavirus vaccine group vs 4.9% of infants in the placebo group developed severe gastroenteritis during the first year of life.^8^ Children presenting with rotaviral diarrhoea aetiology often experience a more severe form of diarrhoea in comparison to children with other diarrhoea.^9^ There is earlier information on the association between rotaviral aetiology and clinical as well as sociodemographic factors. Symptoms such as watery diarrhoea, fever, vomiting and dehydration associated with rotavirus infection can cause further complications that could lead to hospitalisation and other downstream effects.^9^ Socioeconomic factors such as age and crowded households are associated with rotavirus infection.^5^ Maternal illiteracy as well as stunting and wasting was associated with dehydrating rotavirus diarrhoea.^10^ Although there are now point-of-care tests for rotavirus infection, these are often not available in resource-constrained settings.^11^ Therefore, identifying risk factors of rotaviral aetiology would be a useful tool to assist in identifying rotaviral diarrhoea aetiology.

Several factors play a role in increasing the risk of any disease, including rotaviral diarrhoea aetiology. There is not much information on sociodemographic and clinical risk factors for children treated on the outpatient basis, especially in LMIC populations where rotavirus vaccination has been widely distributed. To our knowledge, this is the first analysis to correlate rotaviral aetiology with sociodemographic and clinical factors from several LMICs, especially after rotavirus vaccination. Therefore, this study aims to assess if a rotaviral diarrhoea aetiology in 2–23-month-old children is associated with the child’s earlier vaccination, socioeconomic status and clinical signs and symptoms. We hypothesised that among children presenting with diarrhoea at outpatient clinics, those with a prior receipt of rotaviral vaccine would have a lower probability of rotavirus diarrhoea than unvaccinated children. We also examined the association of rotaviral diarrhoea aetiology with clinical and socioeconomic factors using secondary data from a clinical trial of antibiotics in 2–23-month-old children with diarrhoea and increased risk of mortality carried out in 7 LMICs.

## Methods

### Ethical approval

Ethical approval for the main Antibiotics for Children with Diarrhoea (ABCD) trial was obtained from WHO Ethics review committee (protocol ID: ERC.0002722) as well as institutional review boards from all sites including Bangladesh, India, Kenya, Malawi, Mali, Pakistan and Tanzania.

### Study design and participants

We performed a secondary analysis of a multicentre clinical trial of antibiotics for children with diarrhoea and increased risk of mortality (ABCD, ClinicalTrials.gov Identifier: NCT03130114) ages 2–23 months. ABCD was a multicountry, double-blinded, randomised, placebo-controlled clinical trial that assessed whether a 3-day long treatment with azithromycin would reduce all-cause mortality among 2–23-month-old children with diarrhoea and increased risk of mortality.^12 13^ The ABCD trial was conducted at seven sites including Bangladesh, India, Kenya, Malawi, Mali, Pakistan and Tanzania between June 2017 and July 2019.

The definition of children with diarrhoea and increased risk of mortality in this study was a history of acute watery diarrhoea (>3 loose/watery stool in the past 24 hours) for <14 days with one or more of the following high mortality-risk defining characteristics: moderate acute malnutrition (MAM) (defined as weight-for-length z-score (WLZ) <−2 and >−3 or mid-upper arm circumference (MUAC) >115 mm and <125 mm for children over 6 months), presence of some or severe dehydration, or severe stunting (length-for-age z-score (LAZ) <−3). Children were excluded if they had any of the following: allergy or contraindication to azithromycin; use of antibiotics in the 14 days before presentation or current use of antibiotics; clinical suspicion of *Vibrio cholerae* infection; or living far from the enrolment centre that would hinder direct observation on days 2 and 3; previous or current enrolment in this or any interventional trial; or sibling or another child in the same household enrolled in this trial. Written informed consent was obtained after which the participant was randomised to azithromycin or placebo arm.^13^

Approximately, the first 1000 participants at each site were requested to give a stool sample prior to antibiotic treatment initiation. In this secondary analysis, enteropathogen data obtained from stool samples as well as data collected at baseline were used in order to assess the likelihood of rotavirus aetiology associated with a prior receipt of rotaviral vaccine or various clinical or socioeconomic characteristics of the children.

### Study Variables

The outcome variable in the current association analyses was aetiology of the diarrhoea episode, indicated as rotaviral aetiology (yes/no). The main exposure variable was earlier receipt of at least one dose of rotavirus vaccination. Additional exposure variables of interest included child clinical characteristics including age, high mortality-risk defining characteristic, prolonged duration of diarrhoea, high number of loose/watery stools and low birth weight. Moreover, sociodemographic and environmental exposure variables, including maternal and paternal education, number of under-5-year-old children in the household, presence of animals in the household, quality of sanitary facilities, quality of water sources and wealth quintile of the household, were evaluated. These characteristics have been detailed in online supplemental note 1.

Whole stool samples were collected, and where not possible, a flocked rectal swab was obtained. Diarrhoea-specific aetiology was determined from these samples, frozen at −80°C until analysis, using a quantitative PCR (qPCR) and a customised 85-target TaqMan array card. The details of the qPCR analysis have been described elsewhere^14^ but briefly, using the QIAmp Stool Fast DNA Mini kit (Qiagen, Valencia, CA), total nucleic acid was extracted from the samples, put into the TAC card and run in a ViiA 7 or QuantStudio 7 Flex Real Time PCR system (Thermo Fisher, CA). The method used for determining aetiology cut-offs was adapted from previous large multisite diarrhoea studies.^14^ Briefly, this was based on the quantity of the pathogen DNA/RNA in the stool sample (ie, pathogen burden) at the species level. Each cut-off was determined by calculating the median quantity-specific OR from site-specific models in the previous studies; two large multisite diarrhoea studies: the seven-site GEMS study and the eight-site Malnutrition and the Consequences for Child Health and Development cohort study. Subsequently, the episode-specific attributable fraction (AFe) was computed. A locally estimated scatterplot smoothing regression was then applied, and the highest cycle threshold (Ct) value with an AFe greater than 0.5 was selected as the cut-off for each pathogen. In this analysis, rotavirus aetiology 32.0 Ct cut-off value was used, as per previous studies.^14^

Rotavirus vaccination status was determined by reviewing the child’s vaccination card or, if the card was not available, then queried by caretaker recall. One or more doses of rotavirus vaccine were categorised as yes/no variable for any prior receipt of rotavirus vaccine. Other exposure variables were obtained via questionnaires at baseline by asking the mother/caregiver. Clinical characteristics were determined by clinician assessment at baseline.

### Statistical analysis

We hypothesised that among children presenting with diarrhoea, those with a prior receipt of rotavirus vaccine would have a lower probability of rotavirus diarrhoea than unvaccinated children. Second, we hypothesised that those presenting with low birth weight, longer duration of diarrhoea, more loose/watery stools and being moderately malnourished would have a higher probability of rotaviral diarrhoea aetiology than those without these characteristics. Finally, we hypothesised that those having low maternal and paternal education, lower wealth quintile, poor source of water, no latrine, parents with domestic animal ownership or high number of under-5-year-old children would have a higher probability of rotaviral diarrhoea aetiology in comparison to those without these characteristics.

In order to determine the association between any prior receipt of rotavirus vaccine or selected sociodemographic and clinical factors and rotaviral diarrhoea aetiology, we calculated prevalence ratios (PR) with log-binomial regression models.^15^ If the log-binomial models did not converge, we used log-Poisson models, which provided consistent but not fully efficient estimates of the prevalence ratios and their CIs.^16^ We referred to estimates as prevalence ratios in this study rather than relative risks due to the cross-sectional design of the study. This was done to avoid inferring directionality of the relationship, particularly for clinical factors at presentation which may be a consequence of the diarrhoeal pathogens. Unadjusted (bivariate) as well as adjusted (multivariable) analyses were conducted. The multivariable models encompassed variables that were deemed to be potential confounders for the association between diarrhoeal aetiology and prior rotavirus vaccination.

For statistical analysis, the outcome variable was rotaviral diarrhoea aetiology, categorised as yes/no based on the Ct cut-off values as described above. The main exposure of any prior receipt of rotavirus vaccine was categorised as yes/no. Other variable categorisation details are available in the online supplemental note 1.

We conducted sensitivity analyses to determine the association of clinical factors with rotavirus aetiology after adjusting for rotavirus vaccination as well. We also evaluated the association of sociodemographic factors with rotavirus aetiology after adjusting for clinical factors together with rotavirus vaccination. Furthermore, we evaluated these associations with rotavirus aetiology without a coinfection noted at an aetiological level. This was determined by using the Ct cut-off value for only rotavirus aetiology present in the stool without any coinfection from other aetiology (if any other aetiology was present based on the cut-off, it was considered non-rotaviral aetiology). All statistical analyses were carried out using R version 4.0.2 Software (R Foundation for Statistical Computing).

## Results

**Figure 1:**
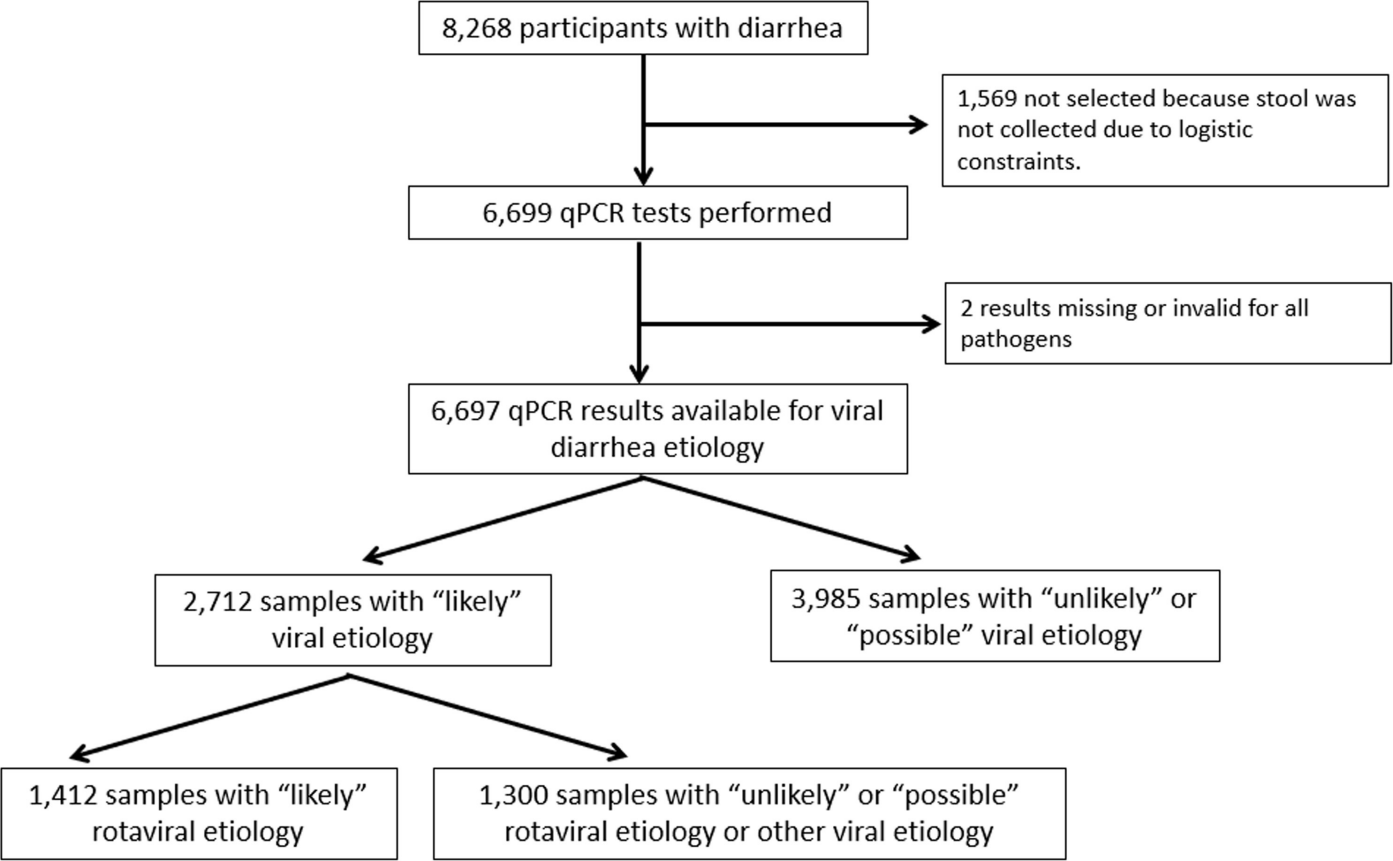
Flow chart of 2–23-month-old children presenting with moderate to severe diarrhoea. ‘Likely’ diarrhoea-associated aetiology was determined by Ct value cut-offs for specific enteropathogens. Ct values greater than these cut-offs but <35 were considered ‘possible’ aetiologies, while Ct value >35 was considered ‘Unlikely’ aetiology. ‘Likely’ rotaviral diarrhoea aetiology was determined by Ct value cut-off of 32.0. Ct, cycle threshold.

All 8268 children aged 2–23-month-old with moderate-to-severe diarrhoea were enrolled in the ABCD trial. Of these, 6,699 provided a stool sample that was analysed with qPCR of which two participants had missing microbiological results. This resulted in 6,697 infants having a result on the microbiological aetiology of the diarrhea episode (figure 1).

The mean age of infants was 11.6 months (SD: 5.3) and 53.8% were male across all sites (table 1). The proportion of children with rotaviral diarrhoea aetiology was 21.1% in the full study population, with lowest proportion at the Kenya (11.0%), Pakistan (11.6%) and Tanzania (11.1%) sites while the highest proportion was in Bangladesh (47.3%) (online supplemental table 1a). The proportion of children with prior receipt of locally available (online supplemental table 1a) rotavirus vaccination was 59.9%, ranging from 1.0%, 2.7% and 7.3% in Asian sites (Bangladesh, India and Pakistan, respectively) and over 99% coverage in Tanzania, Kenya and Malawi (online supplemental table 1a). Each site had a different type of rotavirus vaccination type and number of doses that was administered at the national level (online supplemental table 1b).

**Table 1:** Baseline characteristics of 6697 mothers and their 2–23-month-old children who presented with moderate-to-severe diarrhoea

Baseline characteristics*	Total (n=6697)	Bangladesh (n=1000)	India (n=998)	Kenya (n=1014)	Malawi (n=691)	Mali (n=1000)	Pakistan (n=997)	Tanzania (n=997)
Infant age, months	11.6 (5.3)*	11.2 (5.0)*	11.9 (5.6)*	11.0 (5.7)*	12.0 (4.8)*	12.0 (4.6)*	12.5 (5.5)*	11.0 (5.2)*
Male sex	53.8%	57.8%	52.9%	52.7%	56.9%	53.9%	50.6%	53.2%
Rotavirus vaccination status (any dose >1)	59.9%	1.0%	2.7%	99.6%	99.6%	97.6%	7.3%	99.9%
Length for age Z score	−1.5 (1.4)	−1.8 (1.2)	−2.1 (1.3)	−0.8 (1.3)	−1.6 (1.5)	−1.2 (1.1)	−2.2 (1.3)	−0.8 (1.1)
Weight for length Z score	−1.1 (1.2)	−1.7 (0.9)	−1.4 (1.0)	−0.3 (1.2)	−0.6 (1.2)	−2.0 (0.7)	−1.5 (1.0)	−0.4 (1.3)
Exclusive breastfeeding at time of enrolment	8.7%	1.7%	6.9%	17.9%	4.1%	5.4%	9.2%	14.4%
Maternal education, completed school years	6 (4)	5 (3)	4 (4)	8 (2)	8 (2)	4 (4)	2 (4)	7 (2)
No maternal education	26.0%	18.8%	38.7%	0.9%	0.7%	51.6%	59.1%	4.8%
Maternal age, years	26.0 (5.6)	24.4 (5.2)	26.1 (4.4)	26.0 (5.6)	25.1 (6.0)	26.4 (6.2)	26.8 (5.5)	27.1 (5.8)
Maternal Body Mass Index, kg/m^2^	23.3 (4.7)	22.3 (4.3)	22.6 (4.3)	23.3 (3.8)	23.1 (5.3)	24.0 (4.9)	23.2 (5.0)	24.5 (5.0)
Paternal education, completed school years	6 (4)	6 (4)	5 (4)	10 (2)	10 (2)	4 (5)	3 (4)	8 (2)
No paternal education	24.4%	20.4%	30.9%	0.4%	0.6%	55.1%	52.7%	0.6%
Household wealth above median (wealth quintile)	54.7%	0.1%	71.6%	47.1%	56.6%	92.0%	18.1%	98.4%
Number of children <5 years in the household	1.7 (1.1)	1.2 (0.4)	1.9 (0.9)	1.7 (0.8)	1.4 (0.6)	2.4 (1.8)	2.0 (1.0)	1.3 (0.5)
Proportion of viral aetiology	40.5%	67.2%	35.0%	26.4%	50.7%	41.0%	23.3%	43.2%
Proportion of rotavirus aetiology	21.1%	47.3%	15.9%	11.0%	28.7%	24.4%	11.6%	11.1%

* Numbers indicate mean (SD) for continuous variables and percentages for categorical variables.

The prevalence of rotavirus aetiology was 21.1% in this cohort; 17.6% among participants who had had at least one prior rotavirus vaccination and 25.9% among those who were unvaccinated. Rotavirus vaccination was inversely associated with the prevalence of rotaviral diarrhoea aetiology in unadjusted models (p value <0.001); however, after adjusting for potential confounders, the association between rotavirus vaccination and the prevalence of rotaviral diarrhoea aetiology at presentation was not statistically significant (PR: 0.71, 95% CI 0.49, 1.03) (table 2). Similarly, there was no association of rotavirus vaccination with the prevalence of rotavirus aetiology with no coinfection after adjusting for confounders in sensitivity analyses (online supplemental table 2).

**Table 2:** Association of any earlier dose of rotavirus vaccination with rotaviral diarrhoea aetiology in 2–23-month-old children presenting with acute high-risk non-dysentery diarrhoea based on qPCR cut-offs

Variable	Rotaviral diarrhoea aetiology				
	Prevalence n/N (%)	Unadjusted prevalence ratio (95% CI)	P value	Adjusted* prevalence ratio (95% CI)	P value
Any dose of rotavirus vaccination					
No	647/2491 (25.9%)	Ref.	–	Ref.	–
Yes	656/3724 (17.6%)	0.61 (0.54, 0.69)	<0.001	0.71 (0.49, 1.03)	0.07

* Adjusted for: child age, wealth quintile, mother education, paternal education and site of enrolment.

qPCR, quantitative PCR.

We also examined five clinical correlates of rotavirus aetiology (table 3). Of these, only diarrhoea duration was associated with rotavirus aetiology. The prevalence of rotavirus aetiology was 21.9% in those with shorter duration (0–6 days before presentation) vs 6.3% in those with prolonged diarrhoea (7–13 days) (PR: 2.65; 95% CI: 1.29 to 5.45). No association was noted between any clinical characteristic with the prevalence of a single rotavirus aetiology only (without any other aetiology present as determined by Ct cut-off values) in sensitivity analyses (online supplemental table 3).

**Table 3:** Association of clinical factors with rotaviral diarrhoea aetiology in 2–23-month-old children presenting with acute high-risk non-dysentery diarrhoea based on qPCR cut-offs

Variable	Rotaviral diarrhoea aetiology		
	Prevalence n/N (%)	Adjusted* prevalence ratio (95% CI)	P value
Age (in months)			
2–<6	180/967 (18.6%)	Ref.	–
6–<12	660/2797 (23.6%)	1.00 (0.82, 1.24)	0.93
12–<18	415/1871 (22.2%)	1.00 (0.80, 1.26)	0.96
18–<24	157/1057 (14.9%)	0.84 (0.64, 1.10)	0.21
Risk-defining criterion			
Severe stunting only	144/414 (34.8%)	Ref.	–
Some/severe dehydration only	1099/2835 (38.8%)	1.24 (0.87, 1.77)	0.24
MAM only	962/2225 (35.5%)	1.03 (0.72, 1.48)	0.88
MAM and some/severe dehydration	298/626 (47.6%)	1.44 (0.98, 2.11)	0.06
MAM and severe stunting	141/409 (34.5%)	0.93 (0.57, 1.52)	0.78
Some/severe dehydration and severe stunting	31/95 (32.6%)	1.58 (0.91, 2.73)	0.10
MAM, some/severe dehydration and severe stunting	32/84 (38.1%)	1.24 (0.57, 2.70)	0.59
Duration of diarrhoea (excluding day of enrolment)			
7–13 days of diarrhoea	23/3633 (6.3%)	Ref.	–
0–6 days of diarrhoea	1389/6329 (21.9%)	2.65 (1.29, 5.45)	0.008
Frequency of loose stools (24 hours before enrolment)			
3–6 stools (low frequency)	599/3536 (16.9%)	Ref.	–
>6 stools (high frequency)	813/3156 (25.8%)	1.13 (0.97, 1.32)	0.13
Low birth weight			
No	516/2816 (18.3%)	Ref.	–
Yes	93/363 (25.6%)	1.09 (0.90, 1.32)	0.37
Exclusive breastfeeding at time of enrolment			
No	506/5280 (9.6%)	Ref	–
Yes	79/1412 (5.6%)	0.74 (0.53, 1.03)	0.08

* Model includes all variables in the table plus site of enrolment.

MAM, moderate acute malnutrition; qPCR, quantitative PCR.

Of the seven studied sociodemographic factors, both maternal and paternal education were associated with rotavirus aetiology (table 4). Children whose mothers had at least a secondary education had a lower prevalence of rotavirus aetiology (PR 0.86, 95% CI 0.74, 1.00) as compared with no formal maternal education (table 4). Similarly, those whose fathers had at least secondary education also had a lower prevalence of rotavirus aetiology (PR 0.87, 95% CI 0.75, 1.00) in comparison to those without formal paternal education (table 4). In a sensitivity analysis adjusting for rotavirus vaccination, a potential mediator, there was no association of maternal or paternal education with rotavirus aetiology. There was also no association between any sociodemographic characteristic and the prevalence of rotavirus aetiology without coinfection in sensitivity analyses (online supplemental table 4).

**Table 4:** Association of sociodemographic factors with rotaviral diarrhoea aetiology in 2–23-month-old children presenting with acute high-risk non-dysentery diarrhoea based on qPCR cut-offs

Variable	Rotavirus aetiology		
	Prevalence n/N (%)	Adjusted* prevalence ratio (95% CI)	P value
Maternal education			
No formal education	22.0% (381/1732)	Ref	–
Primary	21.7% (640/2950)	0.92 (0.82, 1.04)	0.20
Secondary	19.3% (331/1719)	0.86 (0.74, 1.00)	0.04
Higher education	20.9% (53/254)	0.88 (0.66, 1.18)	0.39
Paternal education			
No formal education	23.1% (366/1583)	Ref	–
Primary	21.9% (511/2330)	0.94 (0.83, 1.07)	0.33
Secondary	19.6% (411/2094)	0.87 (0.75, 1.00)	0.05
Higher education	20.1% (95/473)	0.90 (0.72, 1.13)	0.38
Wealth quintile			
Q1-poorest	35.5% (307/864)	Ref	–
Q2	27.7% (303/1094)	1.07 (0.94, 1.21)	0.32
Q3	14.9% (160/1071)	0.98 (0.80, 1.20)	0.84
Q4	18.2% (323/1779)	1.18 (0.97, 1.45)	0.10
Q5-richest	16.9% (319/1884)	1.10 (0.89, 1.38)	0.35
Number of children <5 years of age in the household			
1	23.5% (824/3509)	Ref	–
2	18.9% (428/2259)	1.04 (0.93, 1.15)	0.50
>3	17.3% (160/924)	0.99 (0.84, 1.17)	0.94
Presence of animal at home			
No	25.5% (970/3803)	Ref	–
Yes	15.3% (442/2889)	1.05 (0.92, 1.21)	0.47
Presence of improved source of water			
No	12.2% (95/776)	Ref	–
Yes	22.3% (1317/5916)	1.13 (0.88, 1.44)	0.35
Improved sanitation facility			
No	20.9% (285/1365)	Ref	–
Yes	21.2% (1127/5327)	0.92 (0.79, 1.07)	0.30

* Multivariable model includes all variables in the table plus site of enrolment.

qPCR, quantitative PCR.

## Discussion

Among 2–23-month-old children presenting with acute diarrhoea and increased risk of mortality at outpatient clinics, we found a few clinical and socioeconomic factors associated with rotaviral diarrhoea. The association between rotavirus vaccination and the prevalence of rotaviral diarrhoea aetiology was not statistically significant after adjusting for potential confounders. Of the clinical factors, age, risk-defining criteria, frequency of loose stools and low birth weight were not associated with prevalence of rotaviral diarrhoea. Only shorter diarrhoea duration was associated with higher prevalence of rotaviral aetiology. Of the socioeconomic factors, wealth quintile, number of children <5 years of age, presence of animal, improved water and improved sanitation were not associated with prevalence of rotaviral diarrhoea. Only maternal and paternal education was associated with a lower prevalence of rotavirus aetiology.

Our estimates of rotaviral diarrhoea aetiology and the relationship with rotavirus vaccine and sociodemographic and clinical correlates are applicable to children in LMICs with diarrhoea and increased risk of mortality that presented to a facility. The range of the exposure was wide since rotavirus vaccination rates varied between sites (from as low as 1.0% to 99.9%). This heterogeneity could lead to lack of precision and therefore, we adjusted for site of enrolment in all multivariable analyses. In addition, there is likely some degree of maternal recall bias in variables like duration of diarrhoea and number of stools prior to enrolment. This could bias the findings towards the null hypothesis. Nevertheless, our findings suggest that rotaviral aetiology among 2–23-month-old children with acute diarrhoea cannot be reliably determined by data on previous rotavirus vaccination, clinical and sociodemographic factors.

An association between shorter diarrhoea duration and increased prevalence of rotavirus aetiology is consistent with the knowledge that rotavirus causes a more severe form of diarrhoea compared with other viruses, causing children to be sicker and more likely to be dehydrated, leading to hospital presentation.^9^ This is also confirmed by the fact that rotavirus usually has a short incubation period of less than 48 hours.^17^ Results from a cross-sectional study from Nigeria^18^ were consistent with our findings that maternal or paternal secondary education is associated with lower prevalence of rotavirus. It is known globally that maternal education has great impact on child health outcomes^19^ and parent’s education level influences beneficial practices such as uptake of vaccines.

There are some limitations in our study data, including its cross-sectional nature, restricting the cohort to a specific group, the method of determining enteropathogens and not assessing other inflammatory influences or collecting certain variables. First, the nature of this cross-sectional, observational analysis prevents us from making causal inferences. Moreover, the participants in the study included only children with diarrhoea and increased risk of mortality which limits the generalisability of our findings. Using enteropathogen cut-offs is complex, especially within sites where there is high carriage of asymptomatic enteropathogens. Another potential reason behind the poorer response to rotavirus vaccine in LMICs is environmental enteric dysfunction (EED) as suggested in prior studies but not measured in our study.^20^ Due to the parent trial enrolment criteria, we cannot make inferences about rotaviral diarrhoea aetiology in the community or among all children presenting to a facility nor the relationship of factors with diarrhoea incidence or hospitalisation.

The prevalence of rotaviral diarrhoea aetiology in children has been studied widely. However, this unique cohort in children with increased risk of mortality, presenting with diarrhoea at health facilities, provides an opportunity to understand this high-risk group. Most studies focused on the impact on rotaviral diarrhoea aetiology after introduction of rotavirus vaccination, where they noted that after introduction of rotavirus vaccination, the prevalence of rotaviral diarrhoea aetiology is lower. However, this reduction is not as much in low-income settings as it is in high-income settings implying varying vaccine effectiveness globally.^4 6 7 21^ Additionally, malnutrition with micronutrient deficiencies, which is common in low and middle-income settings like those in our study, may play a role in the effectiveness of rotavirus vaccine.^22^ Studies have suggested factors that reduced effectiveness of rotavirus vaccine in such settings include differences in gut microbiota, immaturity of infant immune system and coinfections among others.^23^ Moreover, rotavirus has over 100 different serotypes and rotavirus vaccine covers only some of the most common of them. Therefore, vaccination cannot confer complete protection.^24^ Additionally, association of biomarkers of environmental enteropathy (a subclinical intestinal condition characterised by gut barrier dysfunction, reduced intestinal absorption as well as gut inflammation) is associated with reduced responses to rotavirus vaccination.^25 26^ Conversely, a study noted no associations between EED biomarkers and rotavirus vaccine immunogenicity in rural Zimbabwean infants.^20^ We did not measure EED biomarkers or immunogenicity specifically in this trial and therefore, we cannot explain the reason behind this non-significant association of rotavirus vaccination and rotaviral diarrhoea aetiology.

In summary, in the analysis of this large cohort, we note that a rotaviral aetiology cannot be accurately determined among 2–23-month-old children with acute diarrhoea by information on the child’s earlier vaccination. Receiving any prior rotavirus vaccination does not mean complete protection to rotaviral diarrhoea aetiology among those presenting with diarrhoea especially in an LMIC. Moreover, it is complicated to use characteristics as risk factors of rotaviral diarrhoea aetiology. Although association of risk factors such as shorter diarrhoea duration and maternal and paternal education exists, it may not be very useful in assisting clinical staff in identifying children with rotaviral diarrhoea among all those presenting with diarrhoea at a health facility.

## Supplementary material

10.1136/bmjgh-2024-018337 online supplemental file 1

10.1136/bmjgh-2024-018337 online supplemental table 1

10.1136/bmjgh-2024-018337 online supplemental table 2

10.1136/bmjgh-2024-018337 online supplemental table 3

10.1136/bmjgh-2024-018337 online supplemental table 4

## Data Availability

Data are available upon reasonable request.

## References

[1] UNICEF DATA Diarrhoea. https://data.unicef.org/topic/child-health/diarrhoeal-disease/.

[2] Troeger C, Blacker BF, Khalil IA (2018). Estimates of the global, regional, and national morbidity, mortality, and aetiologies of diarrhoea in 195 countries: a systematic analysis for the Global Burden of Disease Study 2016. Lancet Infect Dis.

[3] Clark A, Black R, Tate J (2017). Estimating global, regional and national rotavirus deaths in children aged. PLoS ONE.

[4] Weldegebriel G, Mwenda JM, Chakauya J (2018). Impact of rotavirus vaccine on rotavirus diarrhoea in countries of East and Southern Africa. Vaccine (Auckl).

[5] Chissaque A, Cassocera M, Gasparinho C (2021). Rotavirus A infection in children under five years old with a double health problem: undernutrition and diarrhoea - a cross-sectional study in four provinces of Mozambique. BMC Infect Dis.

[6] Du Y, Chen C, Zhang X (2022). Global burden and trends of rotavirus infection-associated deaths from 1990 to 2019: an observational trend study. Virol J.

[7] Platts-Mills JA, Amour C, Gratz J (2017). Impact of Rotavirus Vaccine Introduction and Postintroduction Etiology of Diarrhea Requiring Hospital Admission in Haydom, Tanzania, a Rural African Setting. Clin Infect Dis.

[8] Madhi SA, Cunliffe NA, Steele D (2010). Effect of human rotavirus vaccine on severe diarrhea in African infants. N Engl J Med.

[9] Cortese MM, Parashar UD, Centers for Disease Control and Prevention (CDC) (2009). Prevention of rotavirus gastroenteritis among infants and children: recommendations of the Advisory Committee on Immunization Practices (ACIP). MMWR Recomm Rep.

[10] Yeasmin S, Hasan SMT, Chisti MJ (2022). Factors associated with dehydrating rotavirus diarrhea in children under five in Bangladesh: An urban-rural comparison. PLoS One.

[11] Anderson EJ, Weber SG (2004). Rotavirus infection in adults. Lancet Infect Dis.

[12] The ABCD study team (2020). A double-blind placebo-controlled trial of azithromycin to reduce mortality and improve growth in high-risk young children with non-bloody diarrhoea in low resource settings: the Antibiotics for Children with Diarrhoea (ABCD) trial protocol. Trials.

[13] Ahmed T, Chisti MJ, Rahman MW (2021). Effect of 3 Days of Oral Azithromycin on Young Children With Acute Diarrhea in Low-Resource Settings. JAMA Netw Open.

[14] Pavlinac PB, Platts-Mills JA, Liu J (2024). Azithromycin for Bacterial Watery Diarrhea: A Reanalysis of the AntiBiotics for Children With Severe Diarrhea (ABCD) Trial Incorporating Molecular Diagnostics. J Infect Dis.

[15] Wacholder S (1986). Binomial regression in GLIM: estimating risk ratios and risk differences. Am J Epidemiol.

[16] Zou G (2004). A modified poisson regression approach to prospective studies with binary data. Am J Epidemiol.

[17] (2022). Pinkbook: rotavirus. https://www.cdc.gov/vaccines/pubs/pinkbook/rota.html.

[18] Desmennu AT, Oluwasanu MM, John-Akinola Yetunde O (2017). Maternal education and diarrhea among children aged 0-24 months in Nigeria. Afr J Reprod Health.

[19] Health disparities | DASH. https://www.cdc.gov/healthyyouth/disparities/index.htm.

[20] Church JA, Rukobo S, Govha M (2021). Associations between biomarkers of environmental enteric dysfunction and oral rotavirus vaccine immunogenicity in rural Zimbabwean infants. EClinicalMedicine.

[21] Becker-Dreps S, Bucardo F, Vilchez S (2014). Etiology of childhood diarrhea after rotavirus vaccine introduction: a prospective, population-based study in Nicaragua. Pediatr Infect Dis J.

[22] Desselberger U (2017). Differences of Rotavirus Vaccine Effectiveness by Country: Likely Causes and Contributing Factors. Pathogens.

[23] Lopman BA, Pitzer VE, Sarkar R (2012). Understanding reduced rotavirus vaccine efficacy in low socio-economic settings. PLoS One.

[24] O’Ryan M (2009). The ever-changing landscape of rotavirus serotypes. Pediatr Infect Dis J.

[25] Naylor C, Lu M, Haque R (2015). Environmental Enteropathy, Oral Vaccine Failure and Growth Faltering in Infants in Bangladesh. EBioMedicine.

[26] Becker-Dreps S, Vilchez S, Bucardo F (2017). The Association Between Fecal Biomarkers of Environmental Enteropathy and Rotavirus Vaccine Response in Nicaraguan Infants. Pediatr Infect Dis J.

