# Supplementary material for: Is rotavirus aetiology in young children with acute diarrhoea associated with sociodemographic and clinical factors, including rotavirus vaccination status? A secondary cross-sectional analysis of the ABCD trial
Source: BMJ Glob Health. 2025 Jul 27;10(7):e018337. doi: 10.1136/bmjgh-2024-018337 (PMC12306288; doi:10.1136/bmjgh-2024-018337)
Supplement: online supplemental file 1 [file bmjgh-10-7-s001.pdf]

Supplementary note 1. Detail categorization of clinical and sociodemographic variables in this analysis.

Clinical exposures were categorized as follows: age was categorized into 2 - <6 months, 6 - <12, 12 - <18 and 18 - <24 months. High mortality risk defining criterion was categorized into each of the eligibility criteria; severe stunting only, moderate acute malnutrition (MAM) only, some/severe dehydration only, a combination of two of the criteria or a combination of all three. Prolonged duration of diarrhea was defined as 7-13 days in comparison to 0-6 days and frequency of loose stools within 24 hours before enrollment defined as 3-6 stools as low frequency and >6 stools as high frequency of stools. Low birth weight was defined as birthweight of <2500 grams.

In determining the association of socioeconomic factors, maternal and paternal education was categorized to; no formal education, primary, secondary and higher education. These were categorized from number of school years and varied by site. No formal education was 0 years of schooling, primary school was categorized as 6 years of schooling in Mali, 7 years in Tanzania and 8 years in Pakistan, India, Kenya, Bangladesh and Malawi. Secondary was categorized as 4 extra years of school for Tanzania, Pakistan, India, Kenya, Bangladesh and Malawi but 6 years for Mali. Anyone more than those number of years was classified to have higher education. The total number of children under 5 in a household was collected from the baseline questionnaire as categorized as 0, 1, 2 and 3 or more. Presence of animal was determined by checking if the family owned any of the mentioned animals e.g cows, goats, pigs, ducks, chicken, sheep, horses, cats or any other animal.

Improved sanitary facilities and improved water sources were defined based on UNICEF/WHO joint monitoring program (26). Improved sanitary facilities was defined as either one of: flush or pour-flush to piped sewer system, septic tank pit latrines, ventilated-improved pit

latrines, or pit latrines with slab or composting toilets (27). Improved water facilities was defined as either one of: Improved water sources include household connections, public standpipes, boreholes, protected dug wells, protected springs and rainwater collection. Since the question was asked differently at all sites, a yes/no variable was created that included any of these options to determine improved sources. The wealth quintile was also a composite variable which was created by using wealth distribution for each country from the most recent Demographic and Health Survey.
