# Supplementary material for: Is rotavirus aetiology in young children with acute diarrhoea associated with sociodemographic and clinical factors, including rotavirus vaccination status? A secondary cross-sectional analysis of the ABCD trial
Source: BMJ Glob Health. 2025 Jul 27;10(7):e018337. doi: 10.1136/bmjgh-2024-018337 (PMC12306288; doi:10.1136/bmjgh-2024-018337)
Supplement: online supplemental table 1 [file bmjgh-10-7-s002.pdf]

Supplementary table1a. Table showing rotavirus etiology and rotavirus vaccination prevalence by site

	**Rotavirus vaccination status (any dose ≥ 1)**	**Prevalence of Rotavirus etiology**
**Bangladesh (n= 1000)**	1.0%	47.3%
**India (n=998)**	2.7%	15.9%
**Kenya (n=1014)**	99.6%	11.0%
**Malawi (n=691)**	99.6%	28.7%
**Mali (n=1000)**	97.6%	24.4%
**Pakistan (n=997)**	7.3%	11.6%
**Tanzania (n=997)**	99.9%	11.1%

Supplementary table 1b. Detail on the Rotavirus Vaccination used in each of the 7 countries at a country level.

**Country**	**Rotavirus Vaccination type**	**Number of doses**
Bangladesh	(no national immunization program)	-
India	Rotarix (monovalent), Rotateq (pentavalent) and Rotavac (monovalent)	3
Kenya	Rotarix (monovalent)	2
Malawi	Rotarix (monovalent)	2
Mali	Rotateq (pentavalent)	3
Pakistan	Rotateq (pentavalent) and Rotarix (monovalent)	2
Tanzania	Rotarix (monovalent) (25)	2
