# Supplementary material for: Is rotavirus aetiology in young children with acute diarrhoea associated with sociodemographic and clinical factors, including rotavirus vaccination status? A secondary cross-sectional analysis of the ABCD trial
Source: BMJ Glob Health. 2025 Jul 27;10(7):e018337. doi: 10.1136/bmjgh-2024-018337 (PMC12306288; doi:10.1136/bmjgh-2024-018337)
Supplement: online supplemental table 2 [file bmjgh-10-7-s003.pdf]

Supplementary Table 2. Association of any dose of rotavirus vaccination with rotaviral diarrhea etiology only (with no co-infection) in the stools of 2–23-month-old children presenting with acute high-risk non-dysentery diarrhea based on qPCR cut-offs.

**Variable**	**Rotaviral diarrhea etiology with no co-infection**				
**Any dose of Rotavirus Vaccination**	**Prevalence n/N (%)**	**Adjusted[I] prevalence ratio (95% CI)**	**p-value**	**Unadjusted[I] prevalence ratio (95% CI)**	**p-value**
No	460/2491 (18.5%)	Ref	-	Ref	-
Yes	451/3717 (12.1%)	0.76 (0.48, 1.22)	0.26	0.61 (0.53, 0.70)	<0.001

[1] Multivariable model includes: child age, wealth quintile, mother education, paternal education and site of enrolment
