# Supplementary material for: Is rotavirus aetiology in young children with acute diarrhoea associated with sociodemographic and clinical factors, including rotavirus vaccination status? A secondary cross-sectional analysis of the ABCD trial
Source: BMJ Glob Health. 2025 Jul 27;10(7):e018337. doi: 10.1136/bmjgh-2024-018337 (PMC12306288; doi:10.1136/bmjgh-2024-018337)
Supplement: online supplemental table 3 [file bmjgh-10-7-s004.pdf]

Supplementary Table 3. Association of factors with rotaviral diarrhea etiology only after adjusting for confounders in the stools of 2–23-month-old children presenting with acute high-risk non-dysentery diarrhea based on qPCR cut-offs.

**Variable**	**Rotaviral diarrhea etiology with no co-infection**		
	**Prevalence n/N (%)**	**Adjusted[I] prevalence ratio (95% CI)**	**p-value**
**Age**			
2 - <6	128/964 (13.3%)	-	-
6 - <12	462/2,796 (16.5%)	1.00 (0.91, 1.10)	0.95
12 - <18	275/1,867 (14.7%)	0.99 (0.89, 1.10)	0.86
18 - <24	111/1,057 (10.5%)	0.98 (0.86, 1.10)	0.70
**Risk-defining criterion**			
**Severe stunting only**	59/414 (14.3%)	Ref	-
**Some /severe dehydration only**	351/2,825 (12.4%)	1.04 (0.86, 1.27)	0.67
**MAM only**	360/2,223 (16.2%)	0.99 (0.81, 1.21)	0.89
**MAM and some /severe dehydration**	122/625 (19.5%)	1.06 (0.86, 1.33)	0.58
**MAM and severe stunting**	56/409 (13.7%)	1.01 (0.77, 1.32)	0.96
**Some/severe dehydration and severe stunting**	11/95 (11.6%)	1.04 (0.75, 1.42)	0.81
**MAM, some/severe dehydration, and severe stunting**	16/84 (19.0%)	1.08 (0.72, 1.57)	0.72
**Duration of diarrhea (excluding day of enrollment)**			
7-13 days of diarrhea (prolonged)	13/364 (3.6%)	Ref.	-
0-6 days of diarrhea (shorter)	963/6,320 (15.2%)	1.07 (0.89, 1.31)	0.46
**Frequency of loose stools (24 hours before enrollment)**			
3-6 stools (low frequency)	401/3,533 (11.4%)	-	-
>6 stools (high frequency)	575/3,151 (18.2%)	1.01 (0.94, 1.08)	0.83
**Low birth weight**			
No	291/2746 (10.6%)	-	-
Yes	72/426 (16.9%)	1.03 (0.92, 1.14)	0.63

[1] Model includes all variables in the table plus site of enrolment and rotaviral vaccination
