# Supplementary material for: Is rotavirus aetiology in young children with acute diarrhoea associated with sociodemographic and clinical factors, including rotavirus vaccination status? A secondary cross-sectional analysis of the ABCD trial
Source: BMJ Glob Health. 2025 Jul 27;10(7):e018337. doi: 10.1136/bmjgh-2024-018337 (PMC12306288; doi:10.1136/bmjgh-2024-018337)
Supplement: online supplemental table 4 [file bmjgh-10-7-s005.pdf]

Supplementary Table 4 Association of socio-demographic risk factors with rotaviral diarrhea etiology only, after adjusting for confounders in the stools of 2–23-month-old children presenting with acute high-risk non-dysentery diarrhea based on qPCR cut-offs.

**Variable**	**Rotaviral diarrhea etiology with no co-infection**		
	**Prevalence n/N (%)**	**Adjusted[1] prevalence ratio (95% CI)**	**p-value**
**Maternal education**			
No formal education	273/1730 (15.8%)	-	-
Primary	431/2947 (14.6%)	0.97 (0.84, 1.13)	0.72
Secondary	229/1716 (13.3%)	0.96 (0.83, 1.12)	0.62
Tertiary and above	38/254 (15.0%)	0.95 (0.76, 1.18)	0.64
**Paternal education**			
No formal education	261/1581 (16.5%)	-	-
Primary	359/2327 (15.4%)	1.00 (0.84, 1.20)	0.99
Secondary	268/2091 (12.8%)	0.99 (0.83, 1.18)	0.90
Tertiary and above	71/473 (15.0%)	1.00 (0.81, 1.22)	0.98
**Wealth Quintile**			
Q1-Poorest	218/864 (25.2%)	-	-
Q2	206/1093 (18.8%)	0.99 (0.85, 1.16)	0.90
Q3	104/1070 (9.7%)	1.02 (0.96, 1.23)	0.79
Q4	226/1777 (12.7%)	1.03 (0.87, 1.22)	0.75
Q5-Richest	222/1880 (11.8%)	1.03 (0.87, 1.22)	0.75
**Number of children <5 years of age in the household**			
1	571/3503 (16.3%)	-	-
2	299/2257 (13.2%)	1.00 (0.92, 1.08)	0.92
> 3	106/924 (11.5%)	0.98 (0.86, 1.11)	0.70
**Presence of animal at home**			
No	672/3795 (17.7%)	-	
Yes	304/2889 (10.5%)	1.02 (0.90, 1.15)	0.80
**Presence of improved source of water**			
No	67/777 (9.4%)	-	-
Yes	909/5907 (15.4%)	1.03 (0.91, 1.18)	0.62
**Improved sanitation facility**			
No	181/1365 (13.3%)	-	-
Yes	795/5319 (14.9%)	0.99 (0.89, 1.11)	0.89

[1] Multivariable model includes all variables in the table plus site of enrolment, rotaviral vaccination and clinical factors (age, risk defining criteria, duration of diarrhea, frequency of stool and low birth weight)
